# Freeze-Casting of Alumina and Permeability Analysis Based on a 3D Microstructure Reconstructed Using Generative Adversarial Networks

**DOI:** 10.3390/ma17102432

**Published:** 2024-05-18

**Authors:** Xianhang Li, Li Duan, Shihao Zhou, Xuhao Liu, Zhaoyue Yao, Zilin Yan

**Affiliations:** 1 School of Science, Harbin Institute of Technology, Shenzhen 518055, China; 22s058085@stu.hit.edu.cn (X.L.); 23b958017@stu.hit.edu.cn (L.D.); 19b958012@stu.hit.edu.cn (S.Z.); liudong1xh@outlook.com (X.L.); 2 Education Center for Experiment and Innovations, Harbin Institute of Technology, Shenzhen 518055, China; yaozhaoyue@hit.edu.cn

**Keywords:** freeze casting, porous ceramic, microstructure, generative adversarial networks, permeability

## Abstract

In this study, alumina ceramics with hierarchical pores were successfully fabricated using freeze casting. Experimental studies show that both the solid loading of the slurry and the thermal insulation layer at the interface of the slurry and cooling plate can influence the pore characteristics of cast samples. In order to examine the pore characteristics and evaluate the permeability of the freeze-cast samples fabricated under different conditions, a generative adversarial network (GAN) method was employed to reconstruct the three-dimensional (3D) microstructure from two-dimensional (2D) scanning electron microscopy (SEM) images of the samples. Furthermore, GAN 3D reconstruction was validated against X-ray tomography 3D reconstruction results. Based on the GAN reconstructed microstructures, the permeability and pore distribution of the various samples were analyzed. The sample cast with 35 wt.% solid loading shows an optimal permeability.

## 1. Introduction

Porous ceramics are a unique class of ceramics that possess open and percolated pore structures, allowing for the flow of gases and liquids through their solid matrix. Due to their high surface area, thermal stability, and permeability, they find uses in various applications including water purification and filtration [1,2], gas storage [3], thermal insulation [4], artificial bones [5], energy conversion, and storage devices [6]. Porous ceramics can have tailored properties by controlling porosities, pore sizes, shapes, orientations, and distributions. Porous ceramics can be fabricated using several different methods, including the sol–gel method [7], foam template replication method [8], powder sintering with pore formers [9], and freeze casting method [10]. In the freeze-casting method, a ceramic slurry is frozen and then freeze-dried to create a porous structure. The frozen structure is then sintered to form the final porous ceramics. Because of its unique nature, freeze casting is a highly promising technique for the manufacturing of porous ceramics with hierarchical microstructures [10]. These unique straight porous structures make them have great potential for applications in the electrodes in electrochemical devices, such as solid oxide fuel cells [11], solid oxide electrolyzers [12], and lithium batteries [13]. By carefully controlling processing parameters such as cooling temperature, dispersion media, solid loading, and additives like binder and their amount and temperature gradient, diverse microstructures can be achieved [14,15]. Therefore, the formulation of the slurry should take into account all the aforementioned conditions.

For electrochemical device applications, the permeability property is a crucial property that can directly influence the performance of electrochemical devices. Although experiments on microstructural characterization and gas permeation performance of freeze-cast alumina ceramics have been conducted [16], measuring the permeability requires significant efforts. Typically, the permeability experiments were conducted with certain equipment varying by the measuring methods like the steady-state method [17,18] and pulse-test method [19]; both devices measure the permeability by recording the pressure change or the variation in the flow on both sides of inlet and outlet, requiring that the sample has a specific shape and can be placed in the cell of the equipment where the gas flows through. Alternatively, the permeability of the porous media can be assessed through mechanistic model-based numerical simulations in methods like the lattice Boltzmann method (LBM) [20], finite element method (FEM) [21], finite volume method (FVM) [22], and computational fluid dynamics (CFD) [23]. The numerical method relies on accurate 3D microstructure reconstruction of the porous structures. Therefore, obtaining the three-dimensional structure of porous ceramics is crucial for comprehensively studying their properties. Traditional methods for microstructure reconstruction, such as focused ion beam-scanning electron microscope (FIB-SEM) [24] and X-ray computed tomography (XCT) [25], are cost-prohibitive; the FIB-SEM method is especially limited in its ability to reconstruct microstructure exceeding 10s of micrometers. Alternatively, there has been considerable interest in reconstructing the 3D microstructure utilizing 2D SEM images. For example, the stochastic reconstruction algorithm has been applied, showing promising results [26]. However, its low efficiency and extensive computational requirements have limited its applications. With the advancement of machine learning-based image processing technology, it is now possible to utilize these techniques for reconstructing materials’ 3D microstructure [27]. 

In this paper, we explored the fabrication of hierarchically porous alumina ceramics using the freeze-casting method, involving optimizing the slurry formulation and freeze-casting treatments. The effects of slurry’s solid loading and a thermal insulation layer on pore’s characteristics during freeze-casting were investigated. A generative adversarial network (GAN) [28] algorithm was used to reconstruct the anisotropic 3D microstructure with 2D cross-sectional SEM images of the freeze-cast samples for permeability analysis. An XCT reconstruction test was conducted to verify the accuracy of the GAN reconstruction algorithm. Finally, permeability simulations were conducted with Avizo software version 9.0.1 [29] on both GAN- and XCT-reconstructed samples.

## 2. Materials and Methods

### 2.1. Preparation of the Slurry and Freeze Casting

In this study, aqueous alumina slurries were adopted for freeze casting using commercial α-alumina powder (D50 = 0.2 μm, 99.99%, Aladdin, Shanghai, China). Polyacrylic acid (PAA, Macklin, Shanghai, China), polyvinyl alcohol (PVA, Aladdin, Shanghai, China), and glycerol (≥99.5%, Macklin, Shanghai, China) were employed as dispersants, binders, and defoaming agents, respectively, for the slurry making.

**Figure 1:**
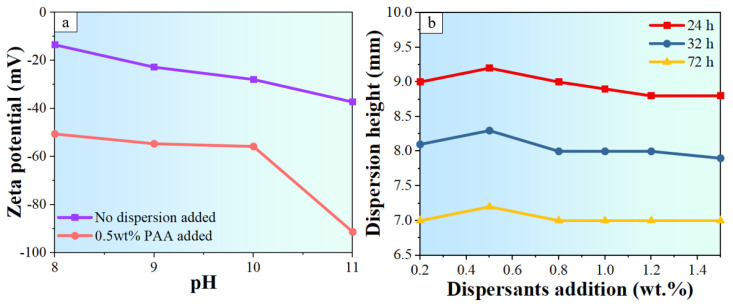
Experiments used to determine slurry formulations: (**a**) Zeta potential test for different pH values and (**b**) sedimentation tests for different amounts of dispersants.

Zeta potential tests were conducted using a Nano-ZS90 zeta meter (Zetasizer, Malvern, Malvern, UK) on a slurry with 0.2 wt.% solid loading to determine the appropriate pH value for achieving optimal dispersion of alumina particles. The pH value of the suspension was tuned by adding 28% ammonia water into it. As shown in Figure 1a, an increase in pH led to an increase in the absolute value of zeta potential, indicating improved dispersion of alumina particles. However, when the pH exceeded 11, the addition of excessive amounts of ammonia water introduced extra water into the slurry. Consequently, the pH of the slurry was set to 11. Sedimentation tests were also performed with different additions of PAA and the slurries were allowed to sediment for 24, 36, and 72 h to observe the sedimentation height. Figure 1b demonstrates that a PAA percentage of 0.5 wt.% resulted in well-dispersed slurry.

**Figure 2:**
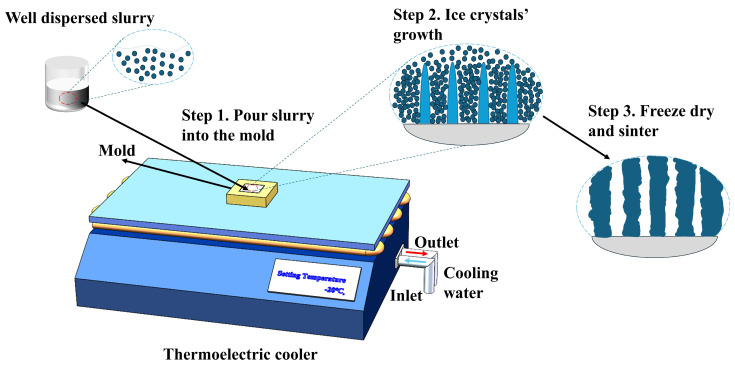
Schematic illustration of the freeze-casting process.

After adding the alumina powder, deionized water, and dispersant, the pH was adjusted to 11. The slurry was then ball milled in a planet ball mill machine (MSK-SFM-1, Hefei Kejing Material Technology, Hefei, China) for 12 h. Subsequently, the binder and an appropriate amount of defoaming agent were added to the slurry and ball milling was carried out for an additional 12 h. After ball milling, the slurry was degassed in a vacuum mixer (TP-1, Bejing Orient Sun-Tec, Beijing, China) for at least 1 h. Subsequently, the slurry was carefully poured into a precisely engineered 5 mm × 5 mm × 5 mm cavity within a 3D-printed ABS (acrylonitrile butadiene styrene) plate of dimensions 10 mm × 10 mm × 5 mm, facilitating the fabrication of a 5 mm × 5 mm × 5 mm cubic freeze-cast sample. Additionally, the mold had a 5 mm thick wall to ensure good lateral thermal insulation and that the temperature gradient occurred solely in the vertical direction. The mold was positioned atop a copper plate fastened to a cold plate of a thermoelectric cooler (TECA LHP-1200CAS, TECA Corp., Chicago, IL, USA), with the temperature set at −20 °C. The entire freeze-casting process is illustrated in Figure 2. Afterward, the mold was placed inside a freeze dryer and underwent vacuum drying (LC-10N-50A, LiChen, Shanghai, China) at a pressure of 3 Pa to 5 Pa and a temperature of −40 °C for a minimum of 36 h, until the sample could be easily peeled away from the mold. The samples were then heated at a rate of 5 °C/min to 500 °C and held at that temperature for 60 min to remove the additives. Finally, the samples were heated to 1400 °C at the same heating rate and held at that temperature for 3 h for sintering; all these procedures were performed in a muffle furnace (KSL-1500X, Hefei Kejing Material Technology, Hefei, China).

Three different sets of cast samples were prepared by following the aforementioned procedures using slurries with solid loadings of 35 wt.%, 40 wt.%, and 45 wt.%. In order to investigate the effect of the thermal gradient at the interface of slurry and cooling plate, another set of samples was fabricated with 35 wt.% solid loading slurry and a thin plastic film insulation layer being placed between the slurry and cooling plate to create a thermal gradient during the freeze casting.

### 2.2. Acquisition of Cross-Sectional SEM Images

**Figure 3:**
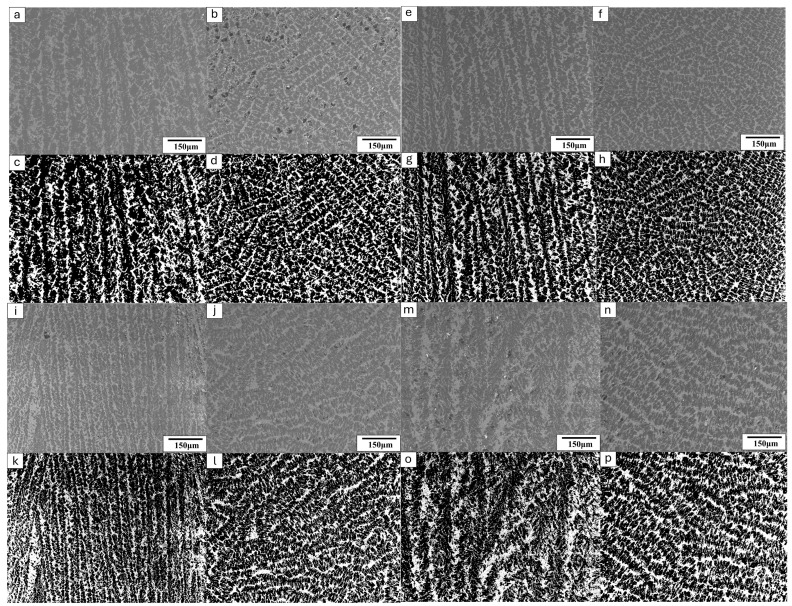
SEM images (150×) of various freeze-cast samples. Grayscale cross-sectional images are displayed for 35 wt.%, 40 wt.%, and 45 wt.% solid loading samples, with images (**a**–**d**), (**e**–**h**), and (**i**–**l**), respectively, representing the samples along and perpendicular to the freezing direction, along with their binarized versions. Additionally, grayscale cross-sectional images of the 35 wt.% solid loading sample with an insulation layer are presented in (**m**,**n**), along with their binarized versions in (**o**,**p**).

**Figure 4:**
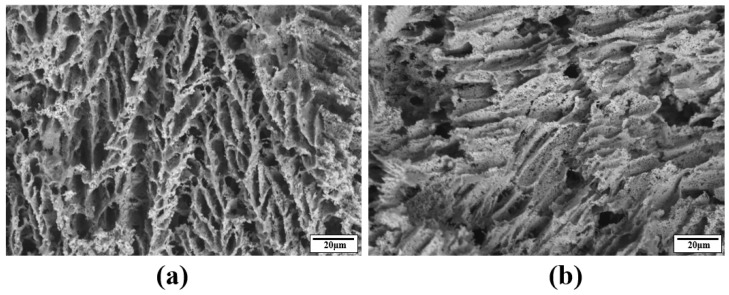
SEM images with higher magnification of a 35 wt.% solid loading sample. (**a**) Along the freezing direction and (**b**) along the direction perpendicular to the freezing direction.

As freeze-cast materials usually have transversely isotropic microstructures, two cross-sectional SEM images are sufficient to capture the microstructures of freeze-cast alumina ceramics. In order to acquire the 2D SEM images, two samples from each set were utilized and the cross-sectional SEM images of the two samples were captured along and perpendicular to the freezing direction, respectively. To prepare the samples for SEM observations, they were immersed in epoxy resin and subjected to vacuum impregnation for a duration of 24 h, ensuring complete resin curing. Subsequently, the samples were polished with sandpaper to expose their cross-sections. Then, a backscatter electron (BSE) detector (TM4000Plus, Hitachi, Tokyo, Japan) was utilized to capture images of these cross-sections. Figure 3 displays the SEM images of typical cross-sections of samples with varying solid loadings. In order to differentiate between alumina and resin, we employed threshold segmentation to binarize the grayscale images. Both the grayscale and binarized SEM images are shown in Figure 3. From the SEM images obtained from the cross-sections along the freeze-casting direction, it can be seen that the microstructure has typical straight pore channels separated by particulate “walls” (Figure 3a,c,e,g,i,k). The “walls” formed during freezing are predominantly parallel to the freezing direction, displaying straight pores between them, except in the case of samples treated with an insulation film. The straight pore features indicate excellent permeability of the samples along the freezing direction. From the SEM images acquired from the cross-sections perpendicular to the freezing direction, it can be seen that the porous microstructure is isotropic in the plane perpendicular to the freezing direction for the samples prepared without an insulation layer treatment (Figure 3b,d,f,h,j,l,n,p). However, for the samples prepared with an insulation film, the walls appear more curved and disorganized (Figure 3m,o) in the freezing direction compared to the other samples. It is also noted that the microcultures in the freeze-cast sample treated with insulation thin film are much coarser than those of samples prepared without an insulation layer. This is attributed to the film’s impact on reducing the temperature gradient along the freezing direction during the freezing process. Higher magnification (1000×) SEM images of the wall microstructures were taken and typical microstructures are shown in Figure 4, with the 35 wt.% solid loading sample as a showcase. Notably, this sample was not impregnated with resin to ensure that the original pores were all preserved for observation. It is shown that the walls are not fully dense but possess some fine pores. However, in order to acquire an adequately large area to represent the entire microstructure for 3D reconstruction, lower magnification SEM images (150×) were used and the fine pores within the walls were missing from the SEM images, resulting in under-estimated porosity by the image method.

### 2.3. Three-Dimensional Microstructure Reconstruction with Two-Dimensional SEM Images

Three-dimensional microstructure was reconstructed using the GAN deep learning algorithm. A typical GAN is composed of two neural networks, namely the generator network and the discriminator network. These two networks are trained in a competitive fashion: the generator creates synthetic data and the discriminator aims to discriminate it. The aim of the GAN is to improve the ability of the generator until the discriminator is no longer able to distinguish the generated synthetic data from the real data. A 3D generative adversarial network was invented to generate a 3D structure from 2D pictures [30]. In their approach, a 200-dimentional latent vector sampled from a probabilistic latent space was mapped to a 64 × 64 × 64 tensor in the generator, representing the reconstruction of the 3D structure. In this study, we used a similar generator to generate a 3D structure and, by changing the hyperparameters of the generator, we can generate a bigger or smaller structure. Notably, the unstable learning problem was avoided by introducing the loss function used in the improved training of Wasstertain GAN (IW-GAN) [31], which has been confirmed to be able to generate higher quality 3D structures compared to the traditional loss function [32]. The loss function is expressed as follows:

(1) $$E_{\tilde{x}\sim p_{g}}[D(\tilde{x})]-E_{x\sim p_{r}}[D(x)]+\lambda E_{\hat{x}\sim p_{\hat{x}}}[{\left(||\nabla_{\hat{x}}D(\hat{x})|{|}_{2}-1\right)}^{2}],$$

where *λ*, *p_g_*, *p_r_*, and $p_{\hat{x}}$, are the gradient penalty, the generator and target distribution, and the distribution sampling uniformly on a straight line between *p_g_* and *p_x_*. *D*(*x*) is the output scaler, which stands for the possibility of *x* being sampled from the training dataset, in which *x* is the real instance input for the discriminator. $\hat{x}$ and $\tilde{x}$ are samples from *p_g_* and $p_{\hat{x}}$, respectively. The loss function is used to measure the difference between generated and real data and provide a goal for optimization.

**Figure 5:**
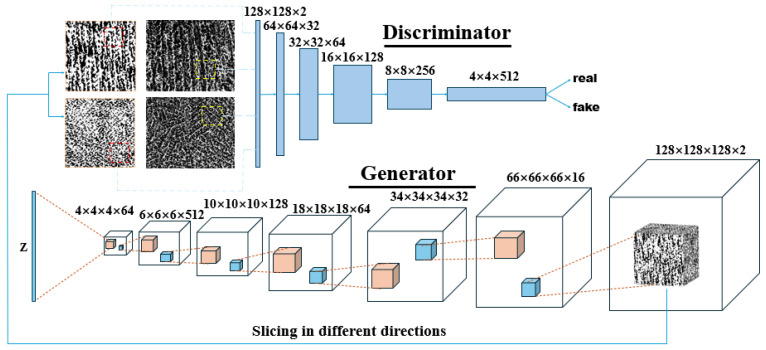
Schematic of the GAN used in this work.

The GAN used in this study is similar to the Slice-GAN used by Kench et al. [33]. Specifically, the GAN used in this work is composed of a 6-layer generator and a 7-layer discriminator. The architecture of this network is demonstrated as Figure 5. In the generator, a 32-dimensional latent vector is fed into it and finally transformed into a 128 × 128 × 128 × 2 tensor, which represents a binarized three-dimensional structure with 128 pixels in three directions, by means of a series of transpose convolutional operations. A softmax layer was used as a classifier of the generator to distinguish pore and solid phases by probability, so that a ‘real’ microstructure with solid and pores can be generated. The hyperparameters for the generator are listed in Table 1. The resulting 3D structure is sliced into 2D images along three different axes and fed into corresponding discriminators. In the discriminator, binarized 2D images from the generator and training set will be discriminated between real and fake. The hyperparameters for the generators are listed in Table 2.

A batch size of 8 was adopted for both the generator and discriminators. The Adam optimizer [34] was used and the Adam optimizer parameters were α = 0.0001, β_1 = 0.9, and β_2 = 0.999. The gradient penalty coefficient is set to λ = 10. Two graphics processing units (GPU) (GeForce RTX 3090, NVIDIA, Santa Clara, CA, USA) were enabled for the acceleration of the training.

### 2.4. X-ray Computed Tomography Reconstruction

**Figure 6:**
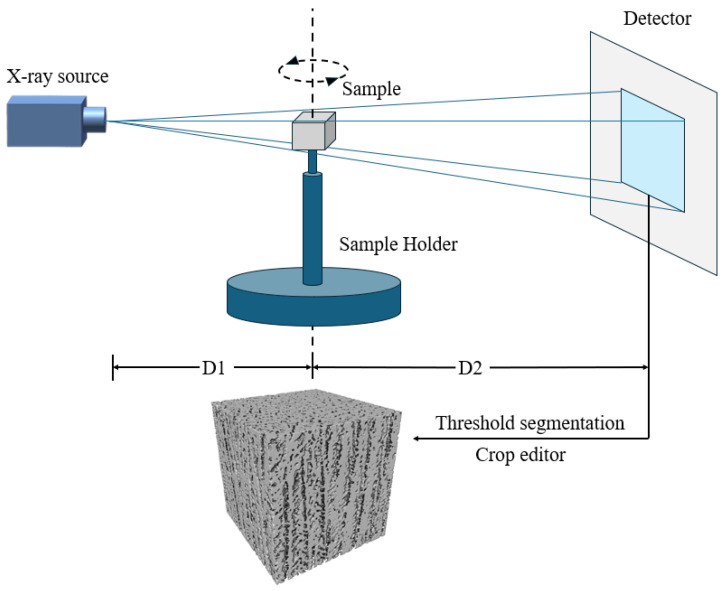
Schematic diagram of the XCT reconstruction.

To evaluate the accuracy of the 3D microstructure reconstructed by the GAN algorithm, X-ray computed tomography (XCT) was employed. A sample with a 35 wt.% solid loading was used for XCT scanning. The source-to-sample distance was D1 = 14.662 mm, while the source-to-detector distance was D2 = 265.000 mm. The sample was scanned using a 40 keV X-ray. The voxel size was 2.802 × 2.802 × 2.802 μm^3^. Binary segmentation and pore analysis were conducted using version 9.1 Avizo software. A cubic region containing 291 × 291 × 291 voxels, approximately 0.512 mm^3^ in size, was extracted from the reconstructed structure. This region size provides sufficient representation of the overall structure, while also expediting the calculation of absolute permeability in subsequent analysis. The schematic diagram of the XCT procedure is depicted in Figure 6. After post-processing, the scanned radiographs were saved as grayscale images and the pore and solid phases were delineated through thresholding.

### 2.5. Permeability Simulation of the 3D Microstructure

**Figure 7:**
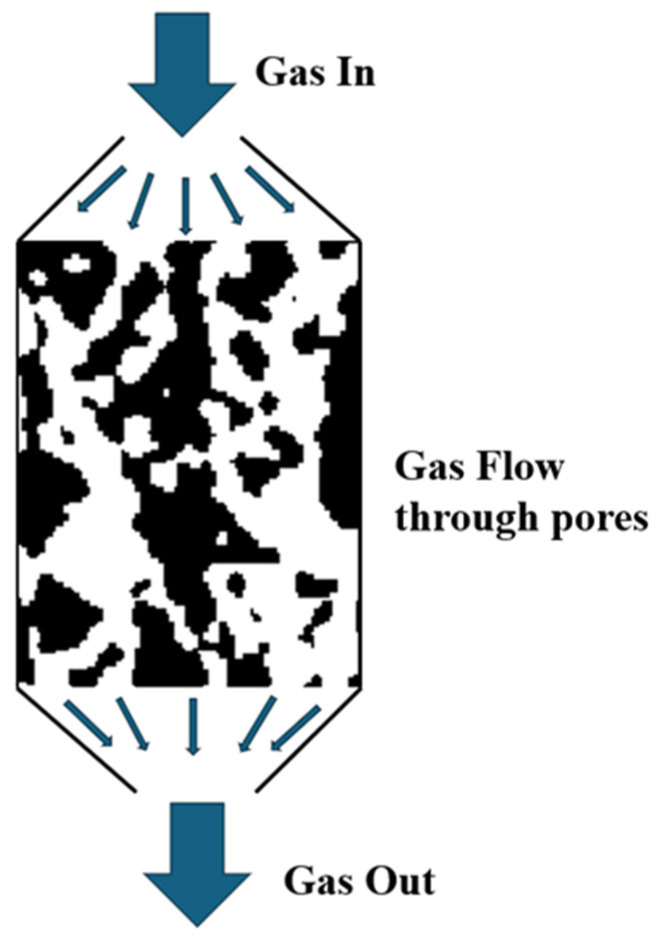
Schematic of Avizo absolute permeability simulation.

To investigate the influence of solid loading on microstructure permeability, we utilized Avizo software for permeability calculation. The setup for permeability test simulation within Avizo is shown in Figure 7. The calculation of absolute permeability was determined using Darcy’s law [35], as shown in Equation (2), as follows:

(2) $$\frac{Q}{S}=-\frac{k}{\mu }\frac{\Delta P}{L},$$

where *Q* is the global flow rate that goes through the microstructure, *S* is the area of the cross-section of the pores that the fluid goes through, *k* is the absolute permeability, *μ* is the dynamic viscosity of the fluid, Δ*P* is the pressure difference applied around the sample, and *L* is the length of the sample in the direction of flow. Once the pressure difference, viscosity, and the reconstructed structure are provided, the absolute permeability can be calculated.

The 3D segmented binary microstructure was used for permeability calculation in Avizo. By setting the pressure difference and the viscosity of the fluid or gas we can acquire the absolute permeability. A typical 10^−5^ viscosity of hydrogen gas was used. The input pressure and the output pressure were set at 20,000 Pa and 10,000 Pa, respectively. The whole calculation was performed on a GPU (GeForce RTX 1660Super, NVIDIA, Santa Clara, CA, USA).

### 2.6. Archimedes Method for Porosity

The porosity of porous material is normally measured by the Archimedes method; since our sample contains mostly open pores, a test method for apparent porosity by boiling water was used to determine the porosity [36]. Firstly, three 35 wt.% solid loading samples were dehydrated at 110 °C for 10 min and then the dry weights were measured. Secondly, samples were hung in the boiling water for 2 h and cooled down to room temperature; then, they were immersed in water for another 12 h and the samples’ weight in water was measured. Lastly, samples were taken out of the water and all drops of water on the surface were wiped off with a moistened cotton cloth and then the saturated weight was measured. The apparent porosity was calculated by Equation (3), as follows:

(3) $$P=\left(\frac{W-D}{W-S}\right)\times 100\%$$

In which *P*, *W*, *D*, and *S* are the apparent porosity, saturated weight, dry weight, and weight in water, respectively. The mean porosity of three 35 wt.% solid loading samples is 76.6%. It should be noted that during our reconstruction with both XCT and GAN methods, the pores in the walls were ignored and they are mainly open pores according to Figure 4. The porosity calculated by the Archimedes method will therefore be very different from that calculated by the image methods (see later Section 3.2).

## 3. Results

### 3.1. Three-Dimensional Reconstruction with the GAN and XCT

**Figure 8:**
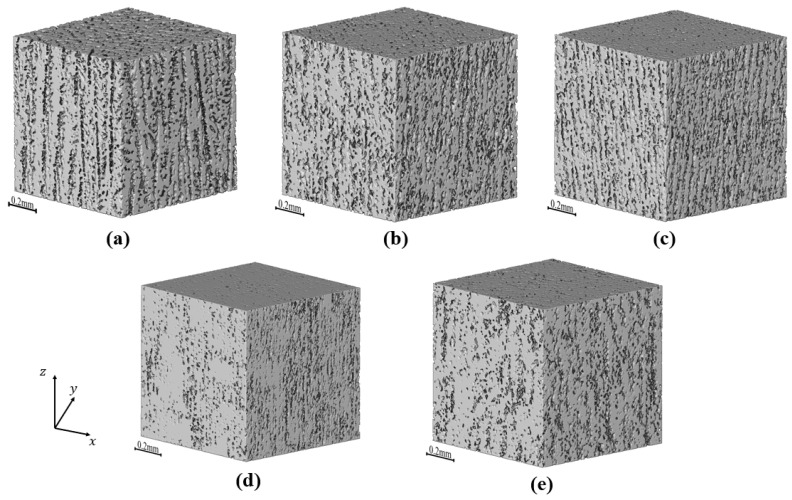
Results of XCT and GAN reconstruction: (**a**) XCT reconstructed sample prepared with 35 wt.% solid loading, (**b**) GAN reconstructed sample prepared with 35 wt.% solid loading, (**c**) GAN reconstructed sample prepared with 40 wt.% solid loading, (**d**) GAN reconstructed sample prepared with 45 wt.% solid loading, and (**e**) GAN reconstructed sample prepared with 35 wt.% solid loading and thermal insulation film treatment.

The GAN reconstructed microstructures have 448 × 448 × 448 voxels, as depicted in Figure 8. The voxel size is 1.82 × 1.82 × 1.82 μm^3^, corresponding to a microstructural volume of approximately 0.512 mm³, equivalent to the size of the XCT reconstruction. It is evident that as the solid loading increases, the size of the pores tends to decrease. However, the visual distinction is not clearly discernible, necessitating the use of a quantitative tool to measure pore shrinkage. From the GAN reconstructed image, we observe orthogonal anisotropic structures that closely resemble those reconstructed by the XCT, along with the presence of wall-like structures. This indicates that the GAN reconstruction can reproduce the realistic anisotropic microstructure from 2D cross-sections of the freeze-cast samples. Further analysis of the GAN reconstruction reveals that as the solid phase content of the slurry increases and the straight pores progressively reduce in size and density. Detailed analysis of specific pores will be discussed later. It should be noted that the pores within the wall structures were ignored due to the resolution of both XCT and SEM images, so the porosity was calculated under the assumption that the walls are dense.

### 3.2. Pore Structure Characterization

**Figure 9:**
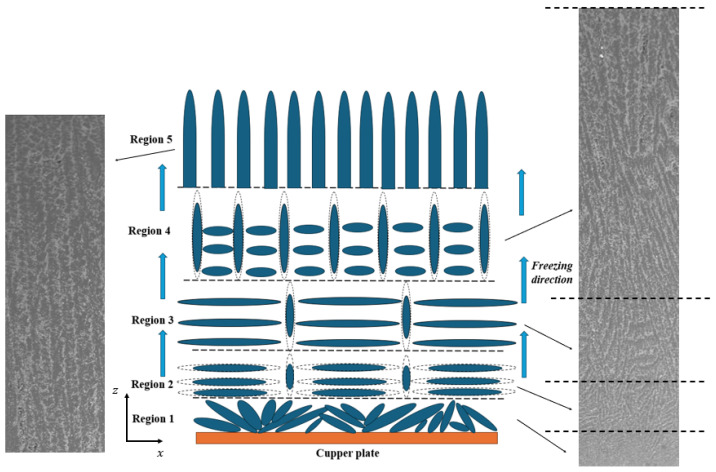
Schematic and SEM images of the ice crystals’ growth.

During the freezing process, the ice growth process can be divided into five regions [37,38], as illustrated in Figure 9. (1) Region 1 represents the bottom part of the sample when the copper surface reaches the frozen temperature, crystals with random orientation began to grow so the microstructure formed here is chaotic and (2) in Region 2, as the crystals grow, the temperature gradient effect becomes more and more important and crystals not only grow along the freezing direction but crystals also grow along the x-y plane. Both vertical and horizontal pores are formed in this region; (3) in Region 3, the horizontal crystals grow preferentially along the temperature gradient; (4) in Region 4, only vertical crystals grow and no horizontal crystals grow and the microstructure becomes more stable; and (5) in Region 5, particle redistribution occurs in the x-y plane and is mastered by the particles fraction only and growth of the crystals achieved a steady state, as demonstrated. The regions we reconstructed are all in the upper part of the samples since the SEM images used for reconstruction were all from this section and represent Region 5.

**Figure 10:**
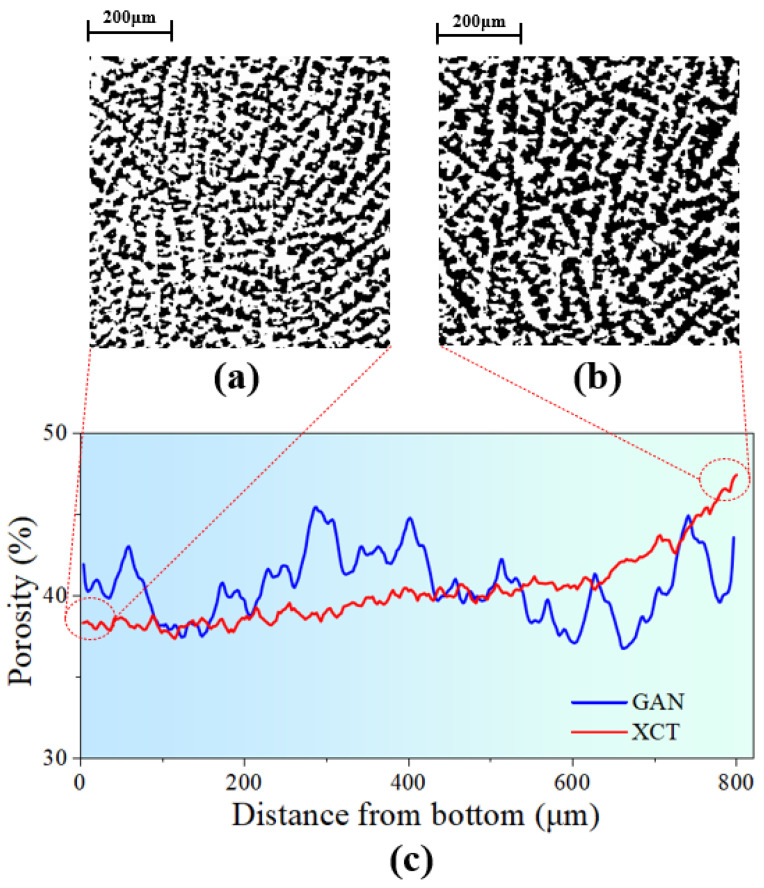
Porosity of the region as analyzed along the freezing direction. (**a**) Binarized images at the bottom of the extracted XCT reconstructed sample, (**b**) binarized images at the top of the extracted XCT reconstructed sample, and (**c**) porosity of GAN and XCT (extracted region) samples along the freezing direction.

Figure 10 shows the areal porosity along the freezing direction. It was found that the porosity differences in the microstructure reconstructed by the GAN and XCT methods were quite small, while there was still a slight increase in the porosity of the XCT sample along the freezing direction. The cross-sectional images of the bottom and top of the extracted XCT sample were also shown in Figure 10a,b, the black phase represents pores and the white phase represents the alumina phase. Pore growth can be easily observed by comparing these two pictures. This is consistent with the ice crystal growth pattern described above.

The porosities of various samples have been precisely quantified based on 3D binary images. For the sample prepared with 35 wt.% solid loading via slurry freeze-casting, the XCT reconstruction (porosity of 41.3%) displays a near-identical porosity to the GAN reconstruction (porosity of 41.4%), indicating that the GAN reconstruction method is capable of accurately capturing the porosity of the freeze-cast alumina samples. Although the image-based porosity is much lower than that measured by the boiling water method, we think the image-based method for porosity measurement can be further improved by acquiring a higher magnification and large area SEM images, which was not in the scope of this study.

Based on the GAN reconstruction, the sample prepared with 35 wt.% solid loading and treated with a thermal insulation film exhibits a reduced porosity of 33%. This indicates that the application of a thermal insulation layer can significantly impact the pore characteristics of the freeze-cast alumina. Based on the GAN-reconstruction, samples freeze-cast with 35 wt.%, 40 wt.%, and 45 wt.% solid loading slurries display porosities of 41.4%, 40.0%, and 38.0%, respectively. This trend suggests that a higher solid loading slurry leads to a more compact structure with fewer pores, resulting in a lower overall porosity.

**Figure 11:**
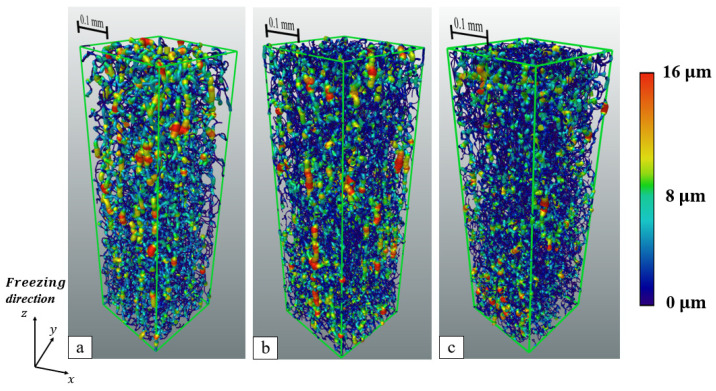
Skeletonized pores of samples prepared with slurry solid loading = 35 wt.% (the intensity of the color represents pore size), (**a**) XCT reconstructed sample (without film treatment), (**b**) GAN reconstructed sample (without film treatment), and (**c**) GAN reconstructed sample (with film treatment).

In order to give a more intuitive representation of the pores in the XCT reconstructed as well as GAN reconstructed samples, we also used Avizo software to skeletonize the pores in the samples and characterize the size of the pores at each area with the width of the skeletonized pores as shown in Figure 11. The thickness of the bones as well as the color shades represent the size of the pores. Since the pores in the structure are very dense, we took 90 × 90 pixels for the XCT sample and 139 × 139 pixels for the GAN sample (approximately 253 × 253 μm^2^) section in the x-y plane, which contains enough straight pores to observe the orientation and size variation in the pores. Figure 11 shows a typical pore network in three samples (XCT and GAN reconstructed 35 wt.% solid loading sample and film treated 35 wt.% solid loading sample reconstructed by GAN).

It can be seen from Figure 11 that the pores of the samples without insulation film treatment show a very clear directionality, with the large pores basically along the freezing direction and very small pores in the horizontal direction. This is reflected in the results of both XCT and GAN reconstructions. For samples padded with an insulation film, the pore distribution is not as ordered as the pores in samples freeze-cast without an insulation film. This is mainly due to the presence of thin film causing the temperature gradient to be reduced, resulting in Region 2 mentioned above being elongated [37], which is obvious both in 2D SEM images and 3D-reconstructed microstructures. However, in comparison to the pores in the XCT and GAN samples, it is seen that there are many fine pores in the GAN reconstructed microstructure; the presence of such fine pores may have an impact on the calculation of permeability. We consider that the presence of these small pores comes from the higher resolution of the SEM shot compared to the XCT scan, which results in the extra small pores existing in the GAN reconstructed microstructure compared to the XCT reconstructed microstructure.

### 3.3. Absolute Permeability Analysis

**Figure 12:**
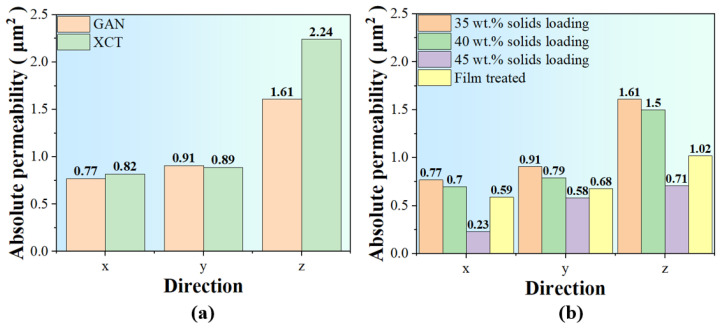
Permeability of different samples: (**a**) permeability of GAN and XCT reconstructed 35 wt.% solid loading sample and (**b**) permeability of 35 wt.%, 40 wt.%, and 45 wt.% solid loading sample and another 35 wt.% solid loading sample treated with film.

In Avizo, we calculated the absolute permeability; the results of a 35 wt.% solid loading’s different directions are depicted in Figure 12a and both XCT and GAN results are demonstrated. It can be seen that the difference in permeability in different directions is obvious and that permeability in the x and y directions (perpendicular to the freezing direction) has a slight difference, which is quite small compared to the permeability along the Z axis (parallel to the freezing direction). As for the difference in permeability of the XCT and GAN samples, the difference could come from the difference in resolution since SEM images have higher resolutions than XCT, meaning structures smaller than XCT’s resolution were ignored, resulting in higher permeability in the XCT sample than the GAN sample. The permeability of samples prepared with different solid loading was also presented in Figure 12b; as the solid loading increased, the permeability decreased and the permeability in different directions shows an anisotropy in each case, while the calculated permeability for the film-treated sample is much smaller than for the unpadded one. However, upon examination, we observe a significant reduction in the permeability of the 45 wt.% solid loading sample across all three directions. Furthermore, there is a substantial increase in the permeability difference between the x and y directions. It is reported by Shanti et al. [39] that a breakthrough concentration lies in the solid loading of slurry. At the breakthrough concentration, the capillary pressure exceeds the osmotic pressure and the solid–liquid interface breaks through the spaces between the concentrated particles, so that no particle redistribution occurs. If the solid loading of slurry is too close to the breakthrough concentration, the growth of ice crystals will be greatly affected [40], which explains the great reduction in absolute permeability of the 45 wt.% solid loading sample with 5 wt.% solid loading increase, compared to the 40 wt.% solid loading sample. 

**Figure 13:**
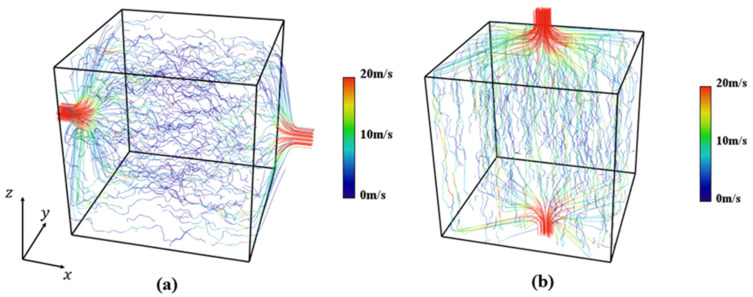
Streamlines and velocity distribution of the fluid inside the 35 wt.% solid loading sample: (**a**) permeability test along the direction perpendicular to freezing direction and (**b**) permeability test along the freezing direction.

Figure 13 shows the streamlines of gas flow in the GAN-reconstructed 35 wt.% solid loading sample with varying colors indicating the magnitude of the flow velocity for permeability test in different directions. It is evident that the streamlines perpendicular to the freezing direction (along the x-axis, as depicted in Figure 13a) exhibit a greater degree of skewness compared to those aligned with the freezing direction (along the z-axis, as depicted in Figure 13b). Furthermore, it is noteworthy that the magnitude of flow velocity in the freezing direction is significantly higher than that in the direction perpendicular to it. These observations serve as direct evidence that the hierarchical porous structure created using the freeze-casting technique is highly effective in enhancing gas transportation along the freezing direction.

## 4. Conclusions

In this study, we devised a precise formulation of alumina slurry for freeze casting of porous ceramics. Freeze casting of alumina was successfully carried out using a homemade apparatus. The freeze-cast alumina sample’s microstructure features hierarchical porous structures with straight pores in the cross-sections perpendicular to the cooling plate and isotropic pores in the cross-sections parallel to the cooling plate. It was found that solid loading of the slurry and thermal gradient design can both affect the microstructure of the freeze-cast alumina ceramics. Based on 2D cross-sectional SEM images of the freeze-cast alumina ceramics, a GAN deep learning algorithm was utilized to reconstruct the 3D microstructure of the freeze-cast ceramics. The comparison between the GAN reconstructed microstructure and the XCT reconstruction has proven that the GAN reconstruction can serve as a low-cost 3D reconstruction method, in contrast to the conventional XCT and FIB-SEM reconstruction, for accurate reconstruction of the isotropic microstructure of the freeze-cast alumina ceramics. Based on GAN-reconstructed 3D microstructures, the permeabilities of the various samples were calculated and compared. Pore analyses were also conducted to determine the pore distribution in the samples, demonstrating that all samples exhibited better permeability along the freezing direction than in the direction perpendicular to it. Absolute permeability calculations revealed that as the solid loading increased, the permeability decreased. A formula with a solid loading of 35 wt.% has proven to be a good formula for the slurry for achieving the best permeability of the freeze-cast alumina ceramics among all the samples we made. Furthermore, the decrease in temperature gradient significantly contributed to the reduction in permeability due to the growth of horizontal ice crystals. The current findings can be a good basis for the fabrication of alumina support with hierarchical pores for the application of solid oxide fuel cells (SOFC)/solid oxide electrolysis cells (SOEC).

## Figures and Tables

**Table 1:** The architecture of the generator.

Layer	Kernel	Stride	Padding	Output Shape
z	-	-	-	4 × 4 × 4 × 64
1	4	2	2	6 × 6 × 6 × 512
2	4	2	2	10 × 10 × 10 × 128
3	4	2	2	18 × 18 × 18 × 64
4	4	2	2	34 × 34 × 34 × 32
5	4	2	2	66 × 66 × 66 × 16
6	4	2	3	128 × 128 × 128 × 2
softmax	-	-	-	128 × 128 × 128 × 2

**Table 2:** The architecture of the discriminator.

Layer	Kernel	Stride	Padding	Output Shape
Input	-	-	-	128 × 128 × 2
1	4	2	1	64 × 64 × 32
2	4	2	1	32 × 32 × 64
3	4	2	1	16 × 16 × 128
4	4	2	1	8 × 8 × 256
5	4	2	1	4 × 4 × 512
6	4	2	0	1 × 1 × 1

## Data Availability

All data supporting the findings of this study are available within the paper and other supporting data are available from the corresponding authors upon request.
